# Small gauge vitrectomy for vitreous amyloidosis and subsequent management of secondary glaucoma in patients with hereditary transthyretin amyloidosis

**DOI:** 10.1038/s41598-020-62559-x

**Published:** 2020-03-27

**Authors:** Shinji Kakihara, Takao Hirano, Akira Imai, Teruyoshi Miyahara, Toshinori Murata

**Affiliations:** Department of Ophthalmology, Shinshu University School of Medicine, 3-1-1 Asahi, Matsumoto, Nagano, 390-8621 Japan

**Keywords:** Eye diseases, Ocular hypertension, Retinal diseases

## Abstract

We conducted a retrospective observational study including 31 eyes of 20 patients in order to investigate the efficacy of 25-gauge vitrectomy for vitreous opacity with minimal conjunctival invasion and subsequent management of intraocular pressure (IOP) secondary to hereditary transthyretin amyloidosis. We followed up these patients for an average of 44.7 ± 32.6 months. The primary outcome was best corrected visual acuity (BCVA) at 1 month after surgery and at the final follow-up visit, with management of subsequent IOP elevation. Secondary outcomes included the post-vitrectomy IOP survival rate, to determine the frequency of IOP elevation requiring glaucoma surgery. Mean age at vitrectomy was 55.4 ± 9.1 years. Logarithm of the Minimum Angle of Resolution (LogMAR) BCVA showed immediate improvement from 0.73 ± 0.62 to 0.00 ± 0.22 at 1 month (*p* = 4.1 × 10^−7^), an improvement that was maintained up to the final follow-up visit, when IOP was maintained at 13.1 ± 5.2 mmHg. The survival rate of post-vitrectomy IOP control was 0.51, 0.38, and 0.23 at 12, 24, and 60 months, respectively. A poor post-vitrectomy IOP survival rate suggests that removing vitreous amyloid via 25-gauge vitrectomy is not sufficient to guarantee good visual function; subsequent careful follow-up and proper glaucoma management is also required in order to achieve this goal.

## Introduction

Familial amyloid polyneuropathies (FAPs) are a group of lethal neurodegenerative diseases that usually result from mutations in the gene encoding transthyretin (TTR) protein^1^. TTR-related FAP, normally referred to as hereditary amyloid TTR (ATTR) amyloidosis^2^, is an autosomal dominant disorder with clinical onset usually before the age of 50 years, and an overall survival time of less than 10 years^3–5^. Hereditary ATTR amyloidosis often includes ocular symptoms such as irregular pupil, dry eye, vitreous opacity, and glaucoma^6–8^. Liver transplantation slows disease progression and increases the survival rate among patients with hereditary ATTR amyloidosis^9,10^. However, as TTR is produced not just in the liver, but also in ocular tissues^11,12^, eyesight deterioration due to vitreous amyloidosis and secondary glaucoma, has become relatively common among patients with hereditary ATTR amyloidosis^6,9,13^.

Visual dysfunction due to vitreous amyloidosis is usually severe, but pars plana vitrectomy (PPV) allows almost complete recovery of visual acuity^14^. Beirão *et al*. reported that 20-gauge PPV significantly improved visual acuity; however, glaucoma increased after such surgery^15^. They also reported that glaucoma diagnosis was more frequent and significantly earlier in patients with complete vitrectomy (extensive vitrectomy, vitreous removal with indentation and shaving of the peripheral vitreous body) than in patients with incomplete vitrectomy (vitreous removal without indentation of the periphery, leaving some peripheral vitreous body). In patients with hereditary ATTR amyloidosis, secondary glaucoma can appear suddenly with a significant increase in intraocular pressure (IOP), leading to severe visual impairment^16^ and even blindness^17^. In many of these patients, it is very difficult to control glaucoma and often multiple glaucoma filtration surgeries are required^18,19^. Therefore, the management of intractable glaucoma is also an important task when performing PPV.

Conventional 20-gauge PPV requires a large conjunctival incision, resulting in postoperative conjunctival and scleral scarring, which is disadvantageous for future filtration surgeries^20^. We have previously reported that 25-gauge PPV allows preservation of the filtration blebs of any previous glaucoma surgery and reduces conjunctiva scar formation, allowing better conditions for possible future filtering surgery^21^. Kawaji *et al*. reported that retinal laser photocoagulation could suppress intraocular TTR production and delay amyloid deposition in ocular tissues^18^.

From these reports, the optimal treatment for vitreous opacities in patients with hereditary ATTR amyloidosis seems to be small-gauge transconjunctival incomplete vitrectomy supplemented with peripheral retinal laser endophotocoagulation. Thereafter, continuous IOP management is vital in order to preserve visual function in hereditary ATTR amyloidosis patients. In the current study, we retrospectively investigated the outcomes of this treatment strategy in hereditary ATTR amyloidosis patients.

## Results

The demographic and clinical characteristics of the study subjects are summarized in Table 1, and the clinical course of all cases is described in Table 2. All patients were Japanese. Mean age at PPV was 55.4 ± 9.1 years. At the time of PPV, five patients (seven eyes) had already been diagnosed with glaucoma; four of these patients (six eyes) had undergone filtration surgery, and the remaining patient (one eye) had not undergone any surgery for glaucoma. The remaining 15 patients (24 eyes) had not previously been diagnosed with glaucoma. Nineteen patients (29 eyes) had the Val30Met variant, and one patient (two eyes) had the Ile84Asn variant of the TTR protein.

**Table 1:** Demographic and clinical characteristics of patients.

Characteristic	Value	No. of eyes
Age at PPV (years)	55.4 ± 9.1	31 eyes
Duration of disease (years)	14.2 ± 5.8	31 eyes
IOP before PPV (mmHg)	15.1 ± 4.4	31 eyes
Time since liver transplantation (years)	13.1 ± 4.6	22 eyes of 14 patients
Glaucoma at the time of PPV	7 eyes of 5 patients	
Pseudopodia lentis	17 eyes of 11 patients	
Pupil irregularities	26 eyes of 17 patients	
Dry eye	20 eyes of 13 patients	
Sex	Male	18 eyes of 10 patients
	Female	13 eyes of 10 patients
Gene mutation	Val30Met	29 eyes of 19 patients
	Ile84Asn	2 eyes of 1 patient
Systemic treatment	Liver transplant	22 eyes of 14 patients
	Administration of tafamidis	7 eyes of 5 patients
	Administration of diflunisal	2 eyes of 1 patient
25-gauge PPV		16 eyes
25-gauge PPV with phacoemulsification and intraocular lens implantation		15 eyes

Data are displayed as mean ± standard deviation. PPV: pars plana vitrectomy; IOP: intraocular pressure.

**Table 2:** Clinical course of all cases.

Case No.	Age at disease onset (years)	Age at liver transplant (years)	Mutation	Laterality	Age at PPV (years)	BCVA at PPV	BCVA at 1 month	BCVA at final visit	IOP at PPV (mmHg)	IOP at final visit (mmHg)	Follow-up period (months)	Times of additional glaucoma surgery
1	40	41	Val30Met	R	56	20/100	20/17	20/50	13	30	22	0
2	62		Val30Met	L	68	20/25	20/13	20/13	9	8	22	0
				R	69	20/50	20/20	20/13	7	8	6	0
3	45	51	Val30Met	R	58	20/500	20/13	20/17	16	13	126	3
4	51		Val30Met	R	58	20/667	20/13	20/22	10	13	96	0
5	41	44	Val30Met	L	56	20/667	20/17	20/13	19	15	36	1
6	58	59	Val30Met	R	63	20/200	20/17	20/17	14	10	99	1
				L	68	20/67	20/17	20/17	17	19	38	0
7	28	30	Val30Met	L	49	20/333	20/13	20/22	15	9	90	1
8	40	42	Val30Met	R	54	20/1000	20/13	20/50	15	13	93	2
				L	56	20/25	20/67	20/17	14	15	63	1
9	35	36	Val30Met	L	52	20/40	20/50	20/20	19	13	84	7
				R	53	20/100	20/20	20/20	16	17	72	2
10	34	38	Val30Met	R	49	20/667	20/13	20/13	16	11	80	2
				L	51	20/33	20/13	20/22	19	15	56	3
11	38	39	Val30Met	L	58	20/100	20/20	20/22	19	10	57	1
				R	60	20/67	20/13	20/17	11	9	24	0
12	58		Val30Met	R	68	20/33	20/17	20/13	11	14	15	0
13	51		Ile84Asn	R	62	20/1000	20/13	20/13	15	9	19	0
				L	62	20/1000	20/13	20/13	9	7	12	0
14	35		Val30Met	L	50	20/500	20/200	20/22	14	25	12	0
15	31	34	Val30Met	R	47	20/13	20/13	20/17	23	12	40	3
				L	49	20/17	20/13	20/13	21	21	18	2
16	63		Val30Met	R	66	20/29	20/17	20/17	17	15	22	1
				L	66	20/33	20/20	20/13	17	9	13	2
17	24	31	Val30Met	L	44	20/67	20/40	20/13	8	9	34	0
				R	46	20/25	20/17	20/17	13	11	12	0
18	47	51	Val30Met	R	60	20/100	20/67	20/1000	24	8	23	0
19	28	30	Val30Met	R	50	20/20	20/17	20/17	21	10	28	1
20	15	27	Val30Met	L	34	20/1000	20/20	20/29	15	18	45	1
				R	35	20/50	20/17	20/33	10	10	28	2

R: right; L: left; PPV: pars plana vitrectomy; BCVA: best corrected visual acuity.

Systemic treatment included liver transplantation for 14 patients (22 eyes), administration of tafamidis^22^ for five patients (seven eyes), and administration of diflunisal^23^ for one patient (two eyes). The time that elapsed between the liver transplant and the vitrectomy was 13.1 ± 4.6 years, and the time that elapsed between disease onset and the vitrectomy was 14.2 ± 5.8 years. On 15 eyes, simultaneous phacoemulsification and intraocular lens implantation was performed.

**Figure 1:**
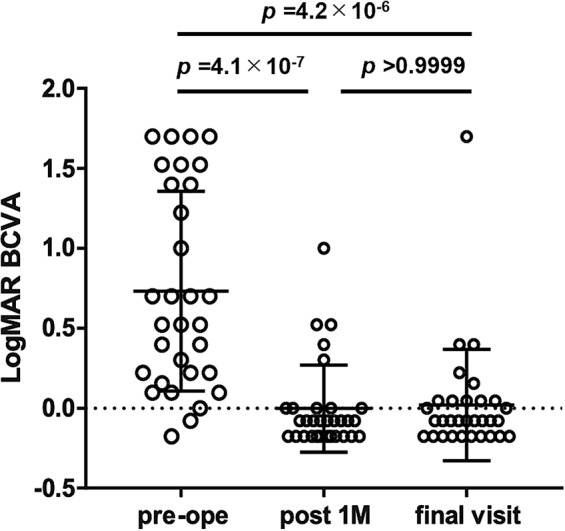
LogMAR BCVA before 25-gauge PPV, at 1 month, and at final visit. 25-gauge PPV significantly improved logMAR BCVA from 0.73 ± 0.62 to 0.00 ± 0.27 immediately, and adequate management of IOP maintained the improved logMAR BCVA. LogMAR BCVA at final visit was 0.02 ± 0.35. These are equivalent to Snellen acuity from 20/107 to 20/20 and 20/19, respectively. Data are displayed as the mean ± standard deviation.

Figure 1 summarizes the results for the primary outcome. LogMAR BCVA significantly and immediately improved from 0.73 ± 0.62 at baseline to 0.00 ± 0.22 after 1 month (*p* = 4.1 × 10^−7^); the improvement was still evident at the final visit (0.02 ± 0.35; *p* > 0.9999 for the difference in logMAR BCVA between 1 month after vitrectomy and the final follow-up visit), because of successful IOP management. These mean values are equivalent to Snellen scores of 20/107, 20/20, and 20/21, respectively. Mean IOP at the final visit was 13.1 ± 5.2 mmHg, with timely and successful glaucoma surgery having been performed where needed. On average, 1.2 ± 1.5 (range: 0–7) such interventions were needed per patient.

**Figure 2:**
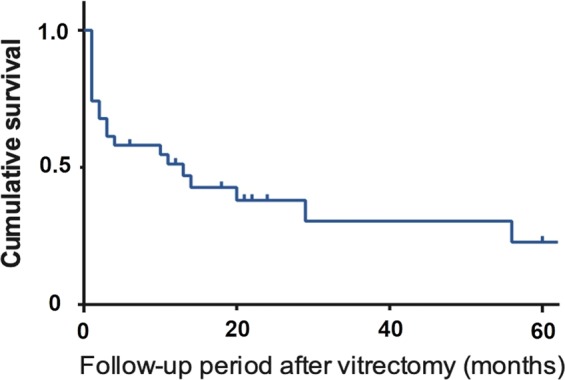
Kaplan-Meier analysis of post-vitrectomy IOP control. Survival criteria: postoperative IOP, ≤ 21 mmHg and ≥6 mmHg. ‘Death’ was determined if survival criteria were not met at two consecutive visits or glaucoma surgery was needed to control IOP.

**Figure 3:**
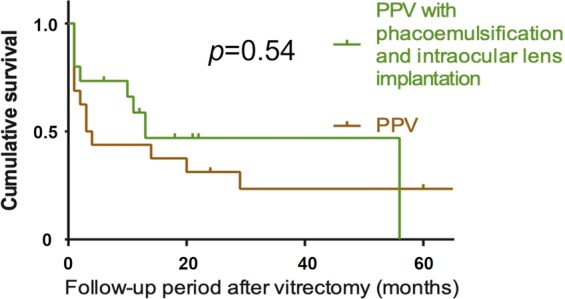
Kaplan-Meier analysis and log-rank test. Comparing between PPV with and without simultaneous phacoemulsification and intraocular lens implantation *(p* = 0.54).

**Figure 4:**
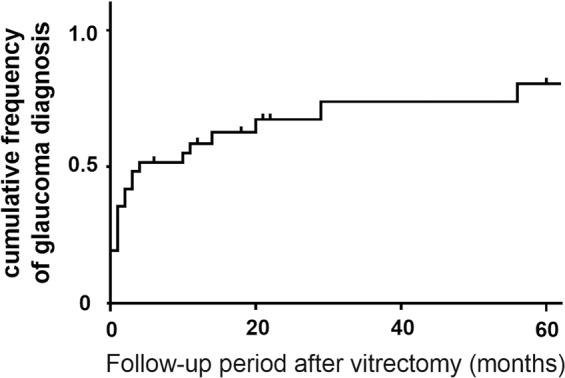
The cumulative frequency of glaucoma after PPV. Glaucoma was diagnosed when the IOP level was ≥22 mmHg, with optic nerve and visual field abnormalities or when glaucoma surgery was needed to control IOP.

**Figure 5:**
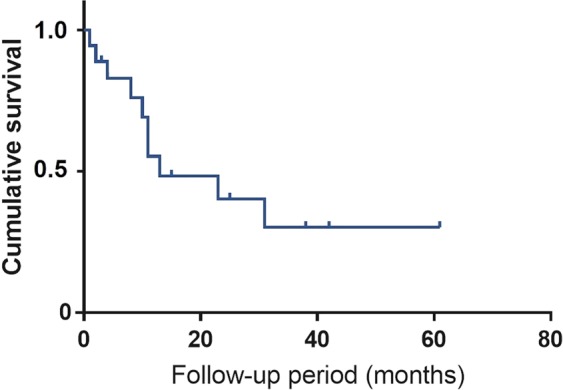
Kaplan-Meier survival curve for the first glaucoma surgery after PPV. Failure was defined as the need for a second glaucoma surgery after PPV.

Figure 2 illustrates the Kaplan-Meier analysis of post-PPV IOP control. The survival rate was 0.51, 0.38, and 0.23 at 12, 24, and 60 months, respectively. Figure 3 illustrates that there is no significant difference between PPV with and without phacoemulsification and intraocular lens implantation in terms of post-operative IOP control (*p* = 0.54). Figure 4 depicts the cumulative frequency of glaucoma diagnosis from the time of 25-gauge PPV. At the time of PPV, the frequency of glaucoma diagnosis was 0.23, while the cumulative frequencies at 1, 3, 6, 12, 24, and 60 months after PPV were 0.39, 0.52, 0.55, 0.58, 0.67, and 0.80, respectively. Eyes with IOP elevation at 1 year after PPV (Group 1) tended to undergo PPV at a younger age (50.9 ± 8.3 years) than did eyes without IOP elevation at 1 year after PPV (Group 2; 59.6 ± 7.9 years; Mann-Whitney test; *p* = 7.8 × 10^−3^). Figure 5 illustrates the Kaplan–Meier survival curve for the first glaucoma surgery after PPV. The survival rates were 0.55, 0.40, and 0.30 at 12, 24, and 60 months, respectively.

### Representative case

At age 34, a female patient began to suffer from persistent diarrhoea (Case 10 in Table 2). Thereafter, she developed symptoms such as numbness and was referred to the Department of Medicine (Neurology and Rheumatology), Shinshu University Hospital, at age 36. There she was diagnosed with hereditary ATTR amyloidosis, with the Val30Met mutation. She received a liver transplant at age 38 and was monitored.

At age 46, she began to experience blurred vision first in her right eye and later in her left eye. At age 49, she was introduced to our department. At the first consultation, BCVA was 20/667 for the right eye and 20/17 for the left eye, and both eyes were diagnosed with vitreous opacity. At that time, intraocular pressure was 22 mmHg in both eyes, but there were no signs of glaucoma. Latanoprost topical eye drops were initiated, upon which IOP dropped to 15 mmHg in both eyes. One month later, 25-gauge PPV and cataract surgery were performed on her right eye.

**Figure 6:**
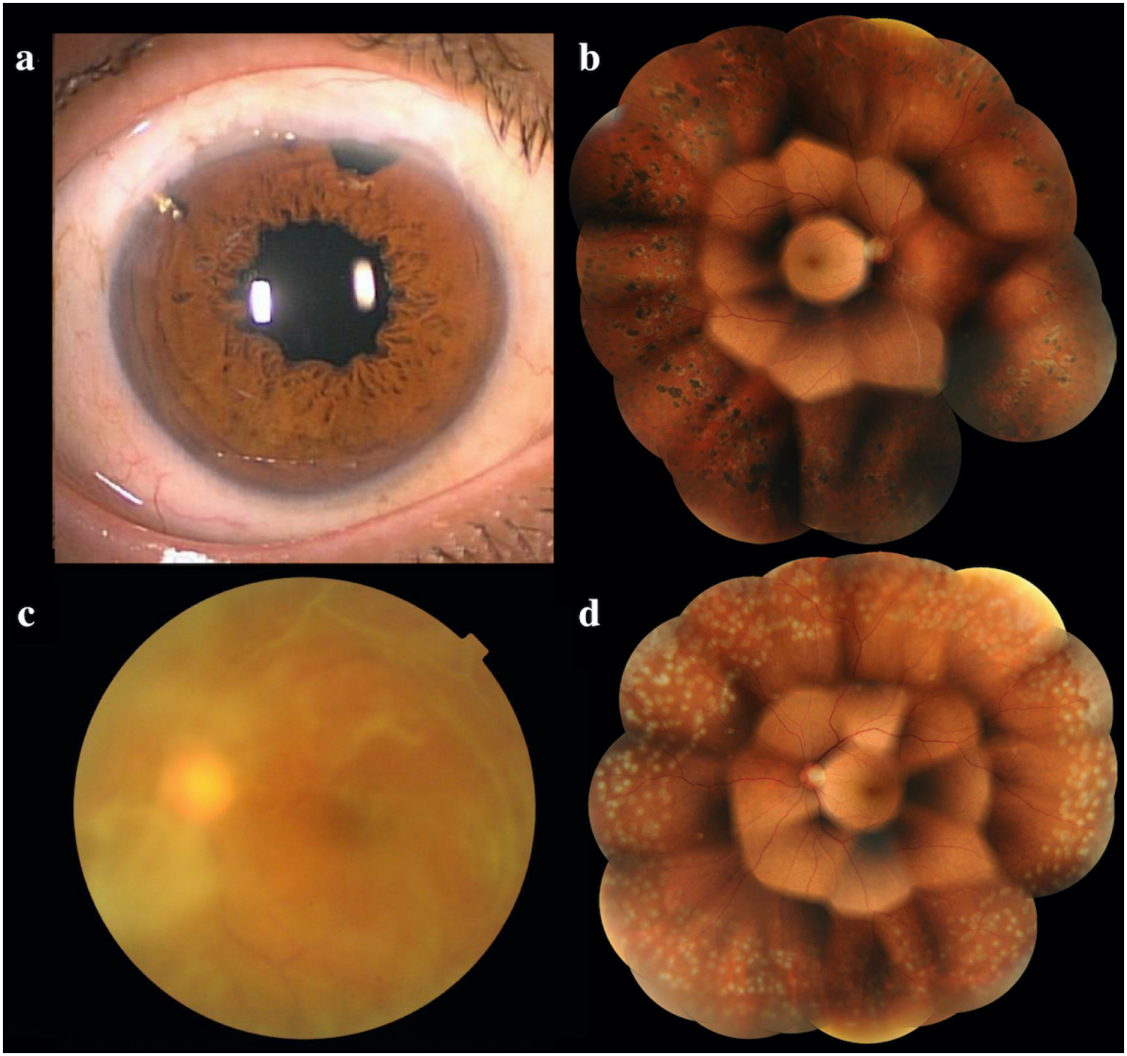
Representative case description (Case 10 in Table 2). (**a**) Anterior segment photography shows characteristic irregularly shaped pupil. (**b**) Montage image at 2 years after vitrectomy. (**c**) Vitreous opacity before vitrectomy. (**d**) Montage image at 3 days after vitrectomy (vitreous opacity remaining in the periphery). (**a, b**) right eye; (**c, d**) left eye.

Following PPV, BCVA of the right eye improved to 20/13, but the IOP gradually rose; despite oral administration of acetazolamide and addition of anti-glaucoma eye drops, IOP rose to 44 mmHg at 3 months after surgery. Ex-PRESS glaucoma filtration surgery was conducted, which temporarily improved IOP. When IOP increased again, trabeculectomy with mitomycin C was performed, 15 months after the vitreous surgery. At the time of the final follow-up visit (80 months after surgery), the right eye had good IOP control (11 mmHg) and maintained good visual acuity (20/13) (Fig. 6a,b).

On the other hand, visual acuity of the left eye gradually deteriorated, and at age 51, 25-gauge PPV and cataract surgery were performed for the left eye as well (Fig. 6c,d). BCVA of the left eye improved from 20/33 to 20/13 immediately after PPV, but IOP increased to 31 mmHg over the next two weeks. One month after PPV, IOP was 35 mmHg and could not be decreased with the addition of oral acetazolamide and anti-glaucoma eye drops. Trabeculectomy with mitomycin C was performed, which temporarily improved IOP. With two further bleb revision procedures where needed, the left eye attained good IOP control (15 mmHg) and maintained good visual acuity (20/22) by the time of the final visit (56 months after surgery).

## Discussion

TTR is a transport protein that carries the thyroid hormone thyroxine^24^, and it exists natively as a tetramer^25^. When TTR is mutated, the tetramers dissociate and the protein misfolds^26^. Surgical invasion is thought to cause diffusion of amyloid fibrils into the eye, especially the trabecular meshwork, which could be the cause of IOP elevation observed after PPV^15^. Occasionally, such IOP elevation leads to blindness^17^. When incomplete PPV is performed, the remaining vitreous body could potentially act as a filter for retaining amyloid fibrils, decreasing amyloid deposition in the trabecular meshwork^15^. Therefore, we conducted incomplete 25-gauge PPV in the current study. BCVA substantially improved from 20/107 to 20/20 when measured after 1 month, but IOP control was relatively poor, with survival rates of 0.51, 0.38, and 0.23, at 12, 24, and 60 months, respectively, suggesting that PPV alone cannot maintain good visual function.

We hypothesized that PPV with cataract surgery could lead to glaucoma development, considering the proximity of the surgical site to the trabecular meshwork. However, the log-rank test indicated no significant link between cataract surgery and glaucoma formation (*p* = 0.54, Fig. 3).

For comparison with a previous report^15^, we calculated the cumulative frequency of glaucoma diagnosis(Fig. 4). In our study, the frequency of glaucoma diagnosis was 0.23 at the time of PPV. The previous report excluded patients that had undergone previous glaucoma surgery, and there were no patients with glaucoma at the time of PPV. This discrepancy may be due to patient selection criteria and the difficulty of diagnosing a patient with glaucoma when vitreous opacity is present and the fundus is blurry. In that study, 24 of 41 eyes (58.5%) had developed glaucoma by 49 months after 20-gauge PPV. In the current study, 73.7% eyes were diagnosed with glaucoma by 49 months after 25-gauge PPV. The difference between previous reports and this study was not significant, even though we preserved a portion of the peripheral vitreous body and performed peripheral scatter retinal endophotocoagulation to reduce TTR production^18^.

In conventional 20-gauge PPV, it is necessary to make a large incision in the conjunctiva, resulting in conjunctiva and sclera scarring, which is disadvantageous if subsequent filtering surgery is needed^20^. 25-gauge PPV can be performed with minimal conjunctival invasion and impact on subsequent filtration surgery^21^.

In this study, 1.2 ± 1.5 instances of glaucoma surgery were needed to control IOP until the final visit (range: 0–7 instances). There were 35 instances of filtration surgery (Ex-PRESS glaucoma filtration surgery, trabeculectomy, bleb revision, needling, and Ahmed Glaucoma Valve implant) and one instance of non-filtration surgery (trabeculotomy ab interno). With longer follow-up, more glaucoma surgery would be required because a high rate of bleb encapsulation leading to IOP elevation has been observed in this disease^19^.

Retina-vitreous specialists often do not perform glaucoma surgery, and vice versa for glaucoma specialists. Consequently, glaucoma specialists and retina-vitreous specialists need to cooperate to provide optimal visual function for hereditary ATTR amyloidosis patients. In this study, the use of 25-gauge PPV with minimal conjunctival damage facilitated subsequent IOP management surgeries, which made it possible to maintain a healthy IOP of 13.1 ± 5.2 mmHg and BCVA of 20/21 long after surgery (44.7 ± 32.6 months).

The present study has several limitations. First, it was designed as a retrospective case series study. Second, all patients were Japanese. Third, the sample size was small, and, in several cases, both eyes were included in the analysis. However, considering the rarity of this disease, the study cohort was relatively large. The particular genetic variants observed in this study are also noteworthy, as the frequency of ocular symptoms has been reported to vary, depending on the TTR variant^14^. Most patients (19) in this study had the Val30Met TTR mutation, which is the most common and well-studied mutation globally. It is also the most common mutation in areas where this extremely rare disease is endemic (e.g. Japan and Sweden)^27^. The results of the current study may be less relevant to areas such as the United States, where other mutations, such as Val122Ile, are more common^28^.

In summary, the IOP survival rate in this study suggests that performing 25-gauge vitrectomy with minimal conjunctival invasion is not sufficient to preserve optimal visual functions. After vitrectomy for vitreous opacity in hereditary ATTR amyloidosis patients, glaucoma filtration surgery is usually necessary; therefore, careful follow-up is required in order to preserve proper visual function. Small gauge vitrectomy for vitreous amyloidosis with minimal conjunctival damage seems to facilitate subsequent glaucoma surgeries and result in better visual function than does conventional 20-gauge vitrectomy.

## Patients and Methods

This retrospective case series study included 20 hereditary ATTR amyloidosis patients (31 eyes) who underwent their first PPV for vitreous opacities between January 2009 and December 2018, whom we followed up for an average of 44.7 ± 32.6 months (range: 6 to 126 months). All patients had their diagnosis confirmed by the Department of Medicine (Neurology and Rheumatology), Shinshu University School of Medicine, and were referred to the Department of Ophthalmology for evaluation of ocular amyloidosis. The gene mutation data, age at disease onset, and age at liver transplant were obtained from their medical history records.

Before PPV, all patients underwent comprehensive ophthalmologic examinations, including measurement of IOP and best-corrected visual acuity (BCVA), slit-lamp biomicroscopy, colour fundus photography, visual field analysis, and optical coherence tomography. Transconjunctival 25-gauge PPV was performed using either the ACCURUS Surgical System or the CONSTELLATION Vision System (Alcon Japan Ltd., Tokyo, Japan), without indentation of the periphery, leaving some peripheral vitreous body. PPV was supplemented with peripheral scatter retinal endophotocoagulation. Phacoemulsification and intraocular lens implantation were also performed for patients with visual acuity loss caused by cataracts.

The primary outcome was BCVA, determined 1 month after surgery and at the final follow-up visit, with proper management of subsequent IOP elevation. If no glaucoma was detected, the follow-up interval, up to the final visit, was approximately once a month. BCVA was converted to logarithm of the Minimum Angle of Resolution (logMAR) BCVA; logMAR BCVA before PPV, at 1 month after surgery, and at the final follow-up visit were compared via two-tailed Dunn’s multiple comparison test. A *p*-value less than 0.05 was considered statistically significant.

The secondary outcome was the post-vitrectomy IOP survival rate, where the frequency of IOP elevation requiring glaucoma surgery was determined. We defined survival based on a previous report: postoperative IOP, ≤21 mmHg and ≥6 mmHg^29^. ‘Death’ was defined as not meeting survival criteria at two consecutive visits, or if glaucoma surgery was needed. We conducted Kaplan-Meier survival analysis for the secondary outcome. To clarify the influence that cataract surgery had on the result, we performed a log-rank test to compare between PPV with and without simultaneous phacoemulsification and intraocular lens implantation. The significant level was set at 0.05. We also calculated the cumulative frequency of glaucoma following PPV in order to compare the result with a previous report^15^. Glaucoma was diagnosed when the IOP level was ≥22 mmHg and optic nerve and visual field abnormalities were present, or when glaucoma surgery was needed to control IOP. To investigate the efficacy of glaucoma surgeries after PPV, we conducted Kaplan–Meier survival analysis for the first glaucoma surgery after PPV. Failure was defined as the need for a second glaucoma surgery after PPV.

We conducted all statistical analysis using GraphPad Prism ver. 7.04 for Windows (GraphPad Software, San Diego, California, USA). We included all possible outliers for purposes of statistical analysis, as they had clinical meaning.

We conducted the study in accordance with the tenets of the Declaration of Helsinki, and the institutional review board of Shinshu University approved the study. Written informed consent was obtained from all patients.

## Data Availability

The datasets generated during and/or analysed during the current study are available from the corresponding author on reasonable request.
